# Common Peroneal Nerve Palsy with Multiple-Ligament Knee Injury and Distal Avulsion of the Biceps Femoris Tendon

**DOI:** 10.1155/2015/306260

**Published:** 2015-05-07

**Authors:** Takeshi Oshima, Junsuke Nakase, Hitoaki Numata, Yasushi Takata, Hiroyuki Tsuchiya

**Affiliations:** Department of Orthopaedic Surgery, Graduate School of Medical Science, Kanazawa University, 13-1 Takara-machi, Kanazawa 920-8641, Japan

## Abstract

A multiple-ligament knee injury that includes posterolateral corner (PLC) disruption often causes palsy of the common peroneal nerve (CPN), which occurs in 44% of cases with PLC injury and biceps femoris tendon rupture or avulsion of the fibular head. Approximately half of these cases do not show functional recovery. This case report aims to present a criteria-based approach to the operation and postoperative management of CPN palsy that resulted from a multiple-ligament knee injury in a 22-year-old man that occurred during judo. We performed a two-staged surgery. The first stage was to repair the injuries to the PLC and biceps femoris. The second stage involved anterior cruciate ligament reconstruction. The outcomes were excellent, with a stable knee, excellent range of motion, and improvement in the palsy. The patient was able to return to judo competition 27 weeks after the injury. To the best of our knowledge, this is the first case report describing a return to sports following CPN palsy with multiple-ligament knee injury.

## 1. Introduction

The posterolateral corner (PLC), which consists of the lateral collateral ligament (LCL), popliteus tendon complex, popliteofibular ligament (PFL), and posterolateral capsule, plays a large role in resisting external rotation of the lateral side of the tibia on the femur [1]. PLC injuries cause severe disability and articular cartilage degeneration. In addition, a multiple-ligament knee injury that includes PLC disruption often causes palsy of the common peroneal nerve (CPN), which occurs in 44% of cases with PLC injury and biceps femoris tendon rupture or avulsion of the fibular head. Approximately half of these cases cannot functionally recover [2].

These factors are all uniquely illustrated in this case, in which the patient sustained a devastating multistructure left knee injury that could have potentially ended his sports career. To the best of our knowledge, this is the first case report describing a return to sports following CPN palsy with multiligament knee injury. This case report aims to present a criteria-based approach to the operation and postoperative management.

## 2. Case Presentation

The patient, a 22-year-old man, experienced a back throw during a game of judo, when his left foot caught on the tatami mat. He sustained a left knee injury resulting from varus and hyperextension force of the knee. He was unable to walk at the scene. Upon presentation to the emergency department, the left knee had large effusions and noticeable tenderness on the posterolateral knee joint. Passive range of motion (ROM) of the injured knee was 115° of flexion, with an extension deficit of 10° (0°–10°–115°). The ROM of the uninjured knee was 5° of hyperextension and 145° of flexion (5°–0°–145°). The patient exhibited a positive Lachman test with a soft endpoint. There was significant laxity (3+) evident during varus stress testing of the left knee at both 0° and 30° of knee flexion, and dial testing was positive at 30° but negative at 90°, when compared to the contralateral side.

**Figure 1:**
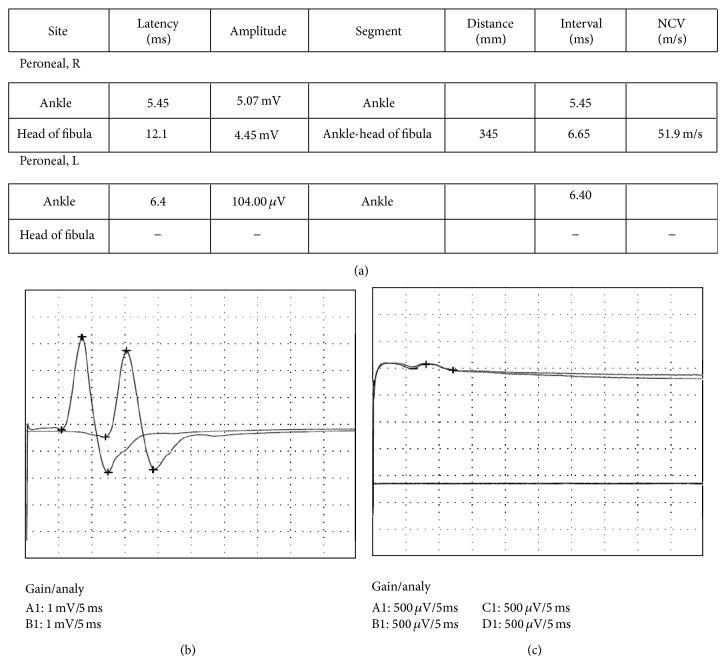
(a) The result of the motor nerve conduction velocity showed the left peroneal nerve palsy. (b) The nerve conduction velocity of the right peroneal nerve. (c) The nerve conduction velocity of the left peroneal nerve. The left peroneal nerve conduction velocity could not be derived 5 days after injury.

The patient could not dorsiflex his left ankle and hallux, and the manual muscle test (MMT) grade of the tibial anterior muscle (TA) and extensor hallucis longus muscle (EHL) was 0, indicating a drop foot, and the sensory function of the CPN area had disappeared. The nerve conduction velocity could not be derived 5 days after injury (Figure 1).

**Figure 2:**
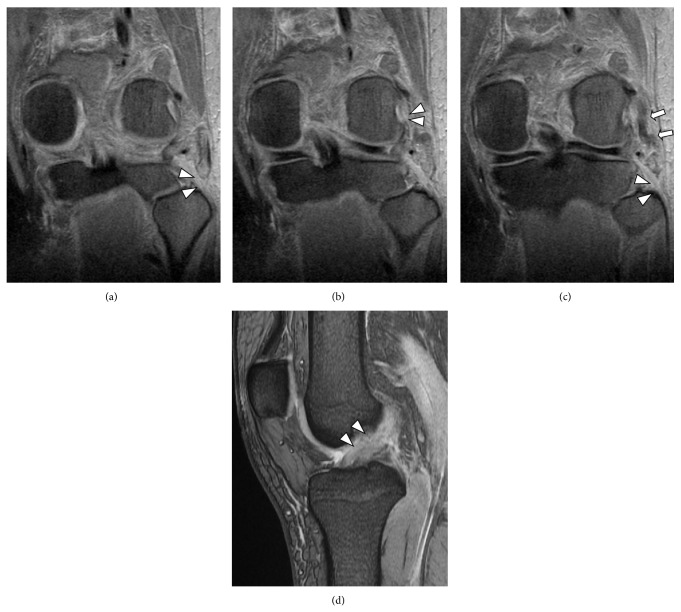
(a) Magnetic resonance imaging (MRI; T2 weighted coronal image) showing the absence of the biceps femoris tendon (arrow head). (b) MRI (T2 weighted coronal image) showing the popliteus muscle tendon rupture at the femoral attachment (arrow head). (c) MRI (T2 weighted coronal image) showing the lateral collateral ligament (LCL) rupture at the fibula head (arrow head) and substance of the LCL (arrow). (d) MRI (T2 weighted sagittal image) showing the anterior cruciate ligament rupture (arrow head).

Radiography showed no fracture in the injured knee. Magnetic resonance imaging showed evidence of a complete anterior cruciate ligament (ACL) rupture, PLC injury that included injury to the popliteus and LCL, and distal rupture of the biceps femoris (Figures 2(a)–2(d)).

A two-staged operation was performed to prevent the loss of ROM owing to arthrofibrosis and the fact that the patient did not require early ACL reconstruction because of the effect of the CPN palsy on activities of daily living (ADL).

**Figure 3:**
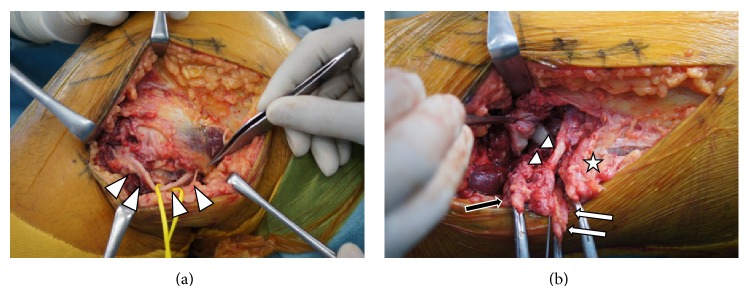
(a) There was evidence of contusion of the common peroneal nerve, and the patient underwent neurolysis (arrow head). (b) There were complete avulsions of the lateral collateral ligament (arrow), popliteofibular ligament (white arrow), and biceps femoris (star) from the fibula head; avulsion of the popliteus muscle tendon from the femoral condyle had also occurred (black arrow).

Following informed consent, the patient underwent surgery 7 days after injury. Examination under general anesthesia revealed a positive Lachman test, positive external recurvatum test, stable posterior drawer test, 3+ laxity with varus stress at both 0° and 30° of knee flexion, and good stability with valgus stress testing. We commenced open repair of the PLC and biceps femoris. A curved incision was made laterally between the femoral condyle and the fibula. Upon splitting the skin and subcutaneous tissue, complete avulsion of the posterolateral structures from both the tibia and fibula became apparent, with complete avulsion of the LCL, PFL, and biceps femoris from the fibula head. Avulsion of the popliteus muscle tendon from the femoral condyle had also occurred. The peroneal nerve was located and inspected. There was evidence of contusion, and the patient underwent neurolysis (Figures 3(a) and 3(b)). Suture anchors were inserted at the attachment of the LCL, PFL, and biceps femoris to the fibular head, and the LCL, PFL, and biceps femoris were sutured.

For the first 3 postoperative weeks, the knee was immobilized with a cast at 30° flexion, and the patient did not bear weight on his surgical leg, to protect the PLC repair from undo forces. After the 3-week cast, the patient advanced to weight bearing, as tolerated, and walking with a soft knee brace and orthosis for the drop foot. The soft knee brace does not immobilize the knee but supports the knee. Adequate quadriceps control for ambulation was achieved at 10 weeks postoperatively, at which time the soft knee brace was taken off for general activity, and the patient's weight-bearing status was progressed to full, unassisted walking. The CPN area gradually recovered after 3 months, and the MMT of the TA and EHL gradually improved 8 months after the first operation.

**Figure 4:**
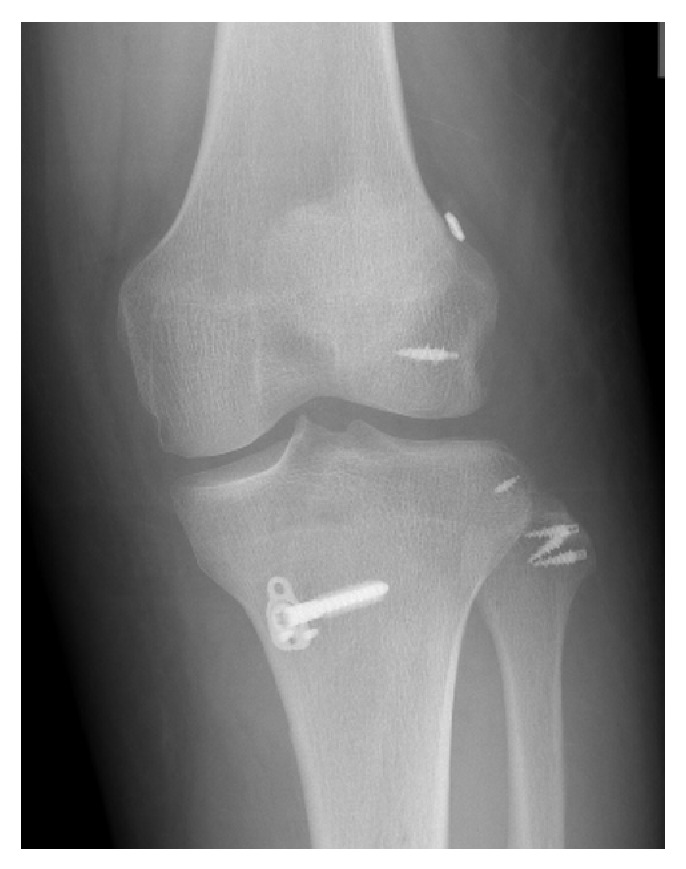
Anatomical single bundle anterior cruciate ligament reconstruction with the ipsilateral semitendinosus tendon was performed.

Nineteen months after the first operation, the MMT of the TA and EHL improved to grades 4 and 1, respectively. There was excellent knee ROM (5°–0°–140°) and good stability with valgus and varus. Because of improved ADL, an anatomical single bundle ACL reconstruction was performed (Figure 4).

**Figure 5:**
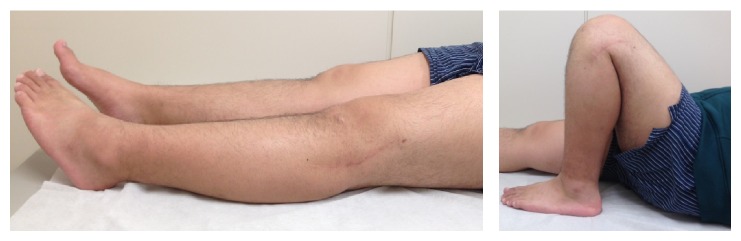
At the final follow-up, 31 months after the injury, there was excellent knee range of motion (5°–0°–145°), stability of the knee, and muscle strength.

Following the ACL reconstruction, the injured knee was progressing favorably. There was excellent knee ROM (5°–0°–145°) and muscle strength, and he returned to competition 27 months after the injury (Figure 5). At the final follow-up, 31 months after the injury, the MMT of the TA and EHL improved to grades 5 and 4, respectively, and sensation in the CPN area had almost completely recovered (Video 1 in Supplementary Material available online at http://dx.doi.org/10.1155/2015/306260). There was good stability with valgus and varus, and the Lachman, pivot shift, and dial tests were negative. At the final follow-up, the subjective International Knee Documentation Committee (IKDC) score was 92.0.

## 3. Discussion

A combined PLC and ACL injury in the knee results from varus and hyperextension forces across the knee [3]. The tension of the biceps femoris increases during the combination of sudden flexion of the hip, varus and hyperextension of the knee, and internal rotation of the leg [4]. The present patient was forced in this position when he experienced a back throw, and eccentric biceps contraction occurred because he chose to endure it. This caused the ACL, PLC, and biceps femoris to rupture spontaneously.

The sciatic nerve branches into the CPN and the tibial nerve at the mid- to distal-third of the thigh, and the CPN descends obliquely over the proximal gastrocnemius muscle and passes lateral to the surgical neck of the fibula. Then, the CPN branches into the lateral sural cutaneous nerve and superficial and deep peroneal nerves distal to the fibular head, at which point it is relatively fixed. At this point, it is vulnerable to stretch forces, such as varus and hyperextension forces that result in ACL and PLC injuries [5].

CPN palsy with multiple-ligament knee injuries and distal avulsion of the biceps femoris tendon is relatively rare. Multiligament knee injuries account for only <0.02% of all orthopedic injuries [6], and 16–40% of these patients suffer an associated injury to the CPN [7]. During multiligament knee injuries, disruption of the PLC is associated with an increased incidence of CPN injury. For example, Bottomley et al. reported that 8 of 18 patients with a PLC injury and biceps avulsions or avulsion-fracture of the fibular head also had CPN palsy [2].

The result of the present case was excellent; the patient had excellent ROM, the knee was stable, and the CPN palsy improved. Two factors potentially explain these results: the early first surgery and the use of a two-staged surgery.

The primary repair was performed within a week of the injury, during which the CPN was located and inspected and underwent neurolysis. For a PLC injury, immediate surgical intervention is reportedly superior to late reconstruction to restore dynamic function of the PLC structures. In primary repair, it is relatively easy to localize the structures, while significant pericapsular scarring makes it difficult to localize and repair discrete structures later than 4–6 weeks after the injury [8].

Acute repair is also required for a biceps femoris rupture. The biceps femoris is the most powerful flexor of the leg; with its biarticular head, it acts as an external rotator and plays a role in hip extension. When it is compromised, flexion strength is reduced by 30–85% [9, 10]. The outcomes with surgical repair of a chronic biceps tendon rupture 4 months after injury have been shown to be poorer than with acute repair [11].

In most cases with multiligament knee injury, nerve traction injury is too extensive to allow direct nerve coaptation. In the presence of CPN motor impairment at the time of an acute repair, neurolysis is needed and is also recommended for CPN palsy if the nerve is not ruptured [7]. However, with a completely transected nerve, the surgeon must decide between direct repair and nerve grafting (most often with a sural nerve autograft). Direct repair is rarely possible in stretch injuries, as the zone of injury must be resected to normal nerve fascicles [12]. However, early PLC repair is less challenging and permits direct visualization of the CPN. Niall et al. reported that, in 14 patients with CPN palsy associated with multiligament knee injury, complete recovery occurred in 3 (21%) patients, partial recovery of useful motor function occurred in 4 (29%) patients, and no useful motor or sensory function returned in the remaining 7 (50%) patients. Furthermore, the prognosis with complete rupture or injuries >7 cm was particularly poor [13].

The two-staged surgery involved primary repair of the PLC and biceps tendon. Following the repair, the cast fixation and non-weight bearing for 3-4 weeks are required [10]. ACL reconstruction that is simultaneously performed in an acutely injured knee, prior to the resolution of swelling, pain, and normalization of movement, has been shown to place an individual at a greater risk for postoperative complications in knee movement. In such cases, two-staged surgery is more appropriate. Ross et al. performed the two-staged surgery, primary repair of PLC and ACL reconstruction, and reported favorite outcome [14]. In addition, in the presence of CPN palsy that affects ADL, early ACL reconstruction is not required.

## 4. Conclusion

To the best of our knowledge, this is the first case report describing a return to sports following CPN palsy with multiple-ligament knee injury. The results of the present case were excellent; the patient had excellent ROM, the knee was stable, CPN palsy improved, and he was able to return to judo. Two factors potentially explain these results: the early first surgery and the use of a two-staged surgery. We believe this case shows better outcomes than previously reported.

## Supplementary Material

At the final follow-up, the MMT of the TA and EHL improved to grades 5 and 4.
